# Oral Tolerance Induction by *Bothrops jararaca* Venom in a Murine Model and Cross-Reactivity with Toxins of Other Snake Venoms

**DOI:** 10.3390/toxins13120865

**Published:** 2021-12-03

**Authors:** Lilian Rumi Tsuruta, Ana Maria Moro, Denise V. Tambourgi, Osvaldo Augusto Sant’Anna

**Affiliations:** 1 Biopharmaceuticals Laboratory, Butantan Institute, São Paulo 05503-900, Brazil; ana.moro@butantan.gov.br; 2 Immunochemistry Laboratory, Butantan Institute, São Paulo 05503-900, Brazil; denise.tambourgi@butantan.gov.br (D.V.T.); osvaldo.santanna@butantan.gov.br (O.A.S.)

**Keywords:** oral tolerance, *Bothrops jararaca*, snake venom, ELISA

## Abstract

Oral tolerance is defined as a specific suppression of cellular and humoral immune responses to a particular antigen through prior oral administration of an antigen. It has unique immunological importance since it is a natural and continuous event driven by external antigens. It is characterized by low levels of IgG in the serum of animals after immunization with the antigen. There is no report of induction of oral tolerance to *Bothrops jararaca* venom. Here, we induced oral tolerance to *B. jararaca* venom in BALB/c mice and evaluated the specific tolerance and cross-reactivity with the toxins of other *Bothrops* species after immunization with the snake venoms adsorbed to/encapsulated in nanostructured SBA-15 silica. Animals that received a high dose of *B. jararaca* venom (1.8 mg) orally responded by showing antibody titers similar to those of immunized animals. On the other hand, mice tolerized orally with three doses of 1 µg of *B. jararaca* venom showed low antibody titers. In animals that received a low dose of *B. jararaca* venom and were immunized with *B. atrox* or *B. jararacussu* venom, tolerance was null or only partial. Immunoblot analysis against the venom of different *Bothrops* species provided details about the main tolerogenic epitopes and clearly showed a difference compared to antiserum of immunized animals.

## 1. Introduction

Snake venoms are composed of a high diversity of proteins and peptides with biological activities, allowing these animals to defend themselves and immobilize their prey [1]. The composition of snake venoms among species displays high variability, both in qualitative and quantitative aspects and complexity [2]. Accidents with snakebite envenoming cause local and systemic effects and represent a public health problem in developing countries, where they reach lower socio-economic segments and kill >100,000 people each year [3]. The primary treatment for the systemic effects of snake envenoming is the intravenous administration of antivenom against specific venoms. Antivenoms specifically neutralize the venoms used in their production and those of related species, which means that antivenoms are produced regionally depending on demand [3]. Indeed, there is a crisis related to the supply of antivenoms, especially in sub-Saharan Africa and parts of Asia; the development of new treatments for patients with snakebite envenoming should be promoted on the basis of recent scientific knowledge related to snake venoms [3]. Recently, several studies have reported that antivenom serum antibodies, generated against specific snake venoms, are cross-reactive with venoms from other species, considering homologous and heterologous snake venoms [4,5,6,7].

Most snakebites in Brazil occur because of the genus *Bothrops* and are considered a serious public health problem. *Bothrops* venom components mainly cause local damage and systemic effects targeting blood hemostasis, endothelial microcirculation, extracellular matrix, and the cardiovascular system [1,8].

Oral tolerance is the induction of peripheral immune tolerance by the oral administration of the antigen and is characterized by the inhibition of the specific immune response to this antigen due to prior oral exposure [9,10,11,12,13,14]. It is a natural and continuous process driven by external antigens. It has a unique immunological importance, as it develops unresponsiveness to ingested food and potential insults from the environment to maintain host homeostasis by protecting against food allergies and colitis caused by autoimmunity [12,13,15]. The gut is regularly exposed to multiple types of antigens, and the associated immune system has specialized immune cells and lymph nodes to balance responses to commensal bacteria (microbiome), innocuous antigens, and harmful microorganisms [11]. Depending on the properties of the antigen, such as size and solubility, the orally administrated antigen that reaches the intestinal epithelium is transported by different routes and can lead to the induction of tolerance or immunity [14]. The oral tolerance induction mechanism has been extensively studied using animal models, mainly for food allergens [11]. It involves multiple factors, and it is known that the dose of the administered antigen and the consumption time are decisive. Administration of a single high dose of antigen leads to the mechanisms of anergy or depletion, whereas exposure to multiple low doses favors the development of regulatory T cells [11,16]. Anergy induction means obtaining antigen-unresponsive T cells, while depletion induction refers to apoptosis of antigen-specific T cells [14]. Previous studies have shown that genetic and environmental factors are involved in the induction of oral tolerance, demonstrating that this characteristic is a process under the influence of multiple factors [17,18,19].

Oral tolerance induction by the administration of one kind of antigen/allergen has been extensively investigated, as has been the mechanism of this process involving immune cells and pathways [11,13,20]. This process has not been explored by the administration of a complex mixture of proteins. Snake envenomation by the oral route does not occur in nature; instead, snakes inject their venoms when there is a dangerous situation and/or they need to defend themselves. Oral antigen application of this kind to induce oral tolerance represents a novel experimental approach. To our knowledge, there is no report of oral tolerance induction using *B. jararaca* venom as an antigen. We propose a method for inducing oral tolerance to *B. jararaca* venom in mice, followed by evaluation of serum cross-reactivity with the toxins from other *Bothrops* species compared to the serum generated by the immunization process.

## 2. Results

### 2.1. Induction of Oral Tolerance by B. jararaca Venom Is Dependent on Dose Administed

**Figure 1:**
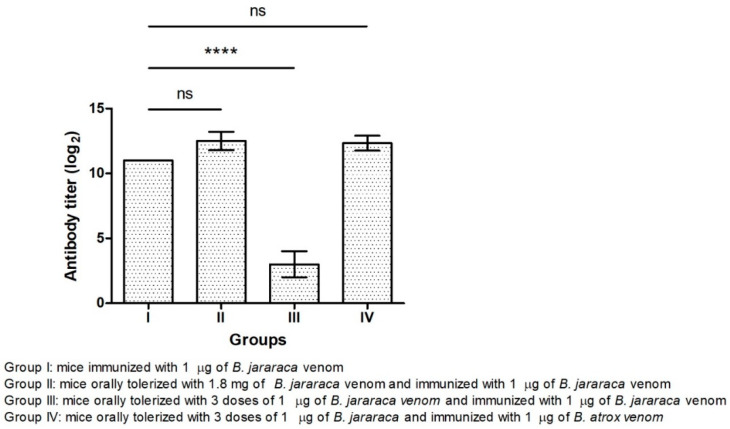
Induction of oral tolerance by administration of *B. jararaca* venom evaluated by antibody titer of mouse antiserum after immunization. Group I was not subjected to oral tolerance. The mice were orally tolerized with a single high dose of venom (Group II) or with low doses of venom on the first, third and fifth day (Group III and IV). Twelve days after receiving the last oral dose, Groups I, II and III were immunized with *B. jararaca* venom, and group IV received *B. atrox* venom. Serum antibody titers corresponding to 35 days after immunization were determined by ELISA. Error bars represent the standard deviation of an experiment (*n* = 3). ANOVA with 95% confidence intervals was used to determine significant differences between each group of mice. ns: not significant; ****: *p*-value was <0.0001.

Oral tolerance is characterized by low IgG levels in the antiserum of animals after immunization with the antigen previously administered orally. Initially, the induction of oral tolerance by administering *B. jararaca* venom was tested using two conditions and the results are summarized in Figure 1. Group I did not receive venom orally. Briefly, one group of mice orally received a single dose of 1.8 mg of the venom (Group II) and other groups received 1 µg of snake venom on the first, third and fifth day (Groups III and IV). At 12 days after the last low dose of 1 µg, all groups were immunized with snake venom combined with silica adjuvant. Groups I, II and III received venom from *B. jararaca*, while Group IV was challenged with venom from *B. atrox*. Antisera corresponding to 7 days after immunization were analyzed, and no difference in antibody titers between all groups of mice was observed (data not shown). Antisera were then collected on the 35th day after immunization and titers determined (Figure 1), followed by analysis based on ANOVA. According to our results, animals that received high doses of *B. jararaca* venom orally were not tolerized (Group II), with antibody titers being similar to those of mice immunized only (Group I). No statistically significant differences were found between Groups I and II and Groups I and IV. Interestingly, mice given a low dose of venom orally and immunized with the same antigen (Group III) had the lowest antibody titer showing the induction of oral tolerance with a statistically significant difference compared to other groups. On the other hand, animals that received a low oral dose of *B. jararaca* and were immunized with *B. atrox* venom (Group IV) showed no tolerance induction. Our results suggest that oral tolerance to snake venom appears to be dose-dependent and is specific for the venom of each snake species.

### 2.2. Characterization of Antisera from Orally Tolerized Animals Reveals Specificity for Other Snake Venoms

**Figure 2:**
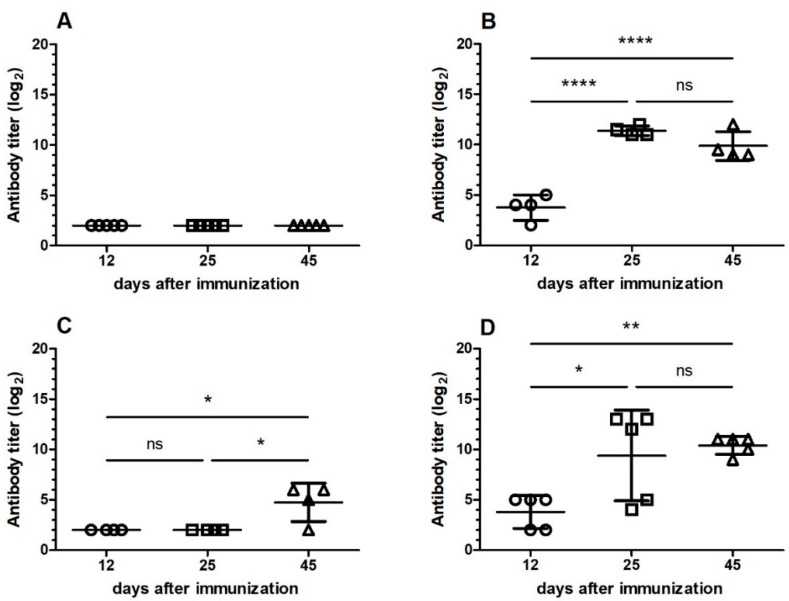
Antiserum specificity for *B. jararaca* venom from mice subjected or not to oral tolerance followed by immunization with snake venom. Microplate wells were coated with 1 µg of *B. jararaca* venom, and serial dilutions of antiserum of Groups I–IV corresponding to 12, 25 and 45 days after snake venom immunization were applied; antibody titer was determined by ELISA. (**A**) Group I: mice tolerized with *B. jararaca* venom and immunized with the same snake venom; (**B**) Group II: mice immunized with *B. jararaca* venom; (**C**) Group III: mice tolerized with *B. jararaca* venom and immunized with *B. jararacussu* venom; (**D**) Group IV: mice immunized with *B. jararacussu* venom. The mean and standard deviation (*n* = 5) of each antibody titer obtained were plotted. ANOVA with 95% confidence intervals was used to determine significant differences between each group of mice group. ns: not significant; *: *p*-value was 0.01–0.05; **: *p*-value was 0.001–0.01; ****: *p*-value was <0.0001.

**Figure 3:**
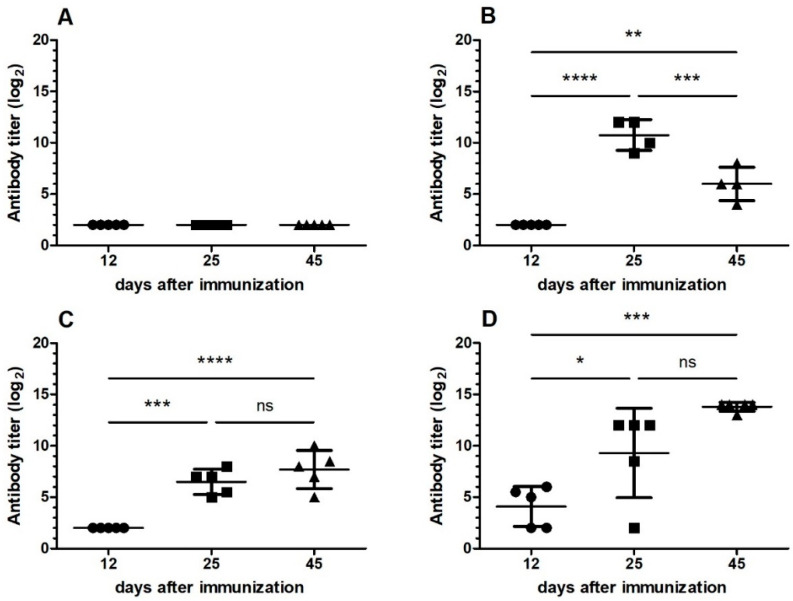
Antiserum specificity for *B. jararacussu* venom from mice subjected or not to oral tolerance followed by immunization with snake venom. Microplate wells were coated with 1 µg of *B. jararacussu* venom, and serial dilutions of antiserum of Groups I–IV corresponding to 12, 25 and 45 days after snake venom immunization were applied; antibody titer was determined by ELISA. (**A**) Group I: mice tolerized with *B. jararaca* venom and immunized with the same snake venom; (**B**) Group II: mice immunized with *B. jararaca* venom; (**C**) Group III: mice tolerized with *B. jararaca* venom and immunized with *B. jararacussu* venom; (**D**) Group IV: mice immunized with *B. jararacussu* venom. The mean and standard deviation (*n* = 5) of each antibody titer obtained were plotted. ANOVA with 95% confidence intervals was used to determine significant differences between each mice group. ns: not significant; *: *p*-value was 0.01–0.05; **: *p*-value was 0.001–0.01; ***: *p*-value was 0.0001–0.001; ****: *p*-value was <0.0001.

To characterize the specificity and cross-reactivity of the antiserum after induction of oral tolerance with *B. jararaca* venom, another experiment was carried out with induction of oral tolerance by administering three doses of 1 µg of the *B. jararaca* venom (Groups I and III). Eight days after the last dose, all groups of mice were immunized intraperitoneally with snake venom combined to adjuvant. Groups I and II were immunized with *B. jararaca*, while Groups III and IV received *B. jararacussu* venom. Antiserum was collected 12, 25 and 45 days after immunization to analyze the specificity of response to *B. jararaca* and *B. jararacussu* venoms. The specificity of antiserum for the venom of *B. jararaca*, using this venom as the antigen is shown in Figure 2, while Figure 3 shows the specificity for the venom of *B. jararacussu.* Our results showed that Group I, orally tolerized and immunized with *B. jararaca* venom, was tolerized for snake venom in view of the low IgG level in all analyzed antisera (Figure 2A and Figure 3A). Group III was tolerized orally and immunized with *B. jararacussu* venom and the antibody titer increased on the 45th day after venom injection when specificity for *B. jararaca* venom was evaluated (Figure 2C), while the same phenomenon was observed on day 25 for the specificity of *B. jararacussu* venom (Figure 3C). Antiserum from groups of mice immunized with snake venom only (Groups II and IV) had high antibody titers for both snake venoms 25 days after immunization (Figure 2B,D and Figure 3B,D). Furthermore, antisera from mice immunized with *B. jararacussu* venom (Group IV) showed reactivity with *B. jararaca* venom (Figure 2B) and antisera from mice immunized with *B. jararaca* venom (Group II) showed reactivity with *B. jararacussu* venom (Figure 3B). In the case of specificity for *B. jararacussu* venom, mice immunized with *B. jararaca* venom (Group II) showed a reduction in antibody titer 45 days after immunization (Figure 3B).

**Figure 4:**
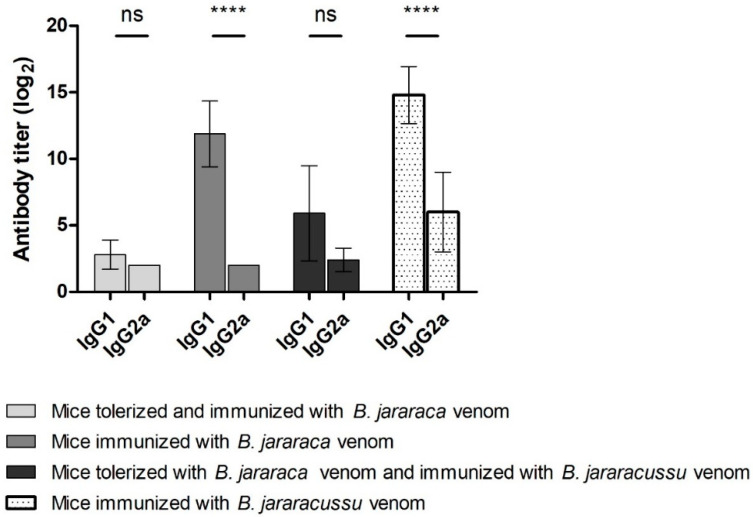
IgG1 and IgG2a antibody titers of groups of mice subjected to oral tolerance induction with *B. jararaca* venom followed by immunization. Microplate wells were coated with 1 µg of *B. jararaca* venom, and serial dilutions of Groups I-IV antiserum, as previously described in “Section 4”, corresponding to 45 days after immunization with snake venom, were applied; antibody titer was determined by ELISA using mouse anti-IgG1 or anti-IgG2a antibodies conjugated to HRP. Error bars represent the standard deviation of one experiment (*n* = 5). 2-way ANOVA with 95% confidence intervals was used to determine significant difference between IgG1 and IgG2a antibody titers for each group. ns: not signigficant; ****: *p*-value was < 0.0001.

IgG1 and IgG2a titers were determined for antisera obtained 45 days after immunization for Groups I, II, III and IV (Figure 4). Orally tolerized mice had lower antibody titers than immunized mice and no difference between IgG1 and IgG2a titers was observed. IgG1 antibody titers from mice immunized with snake venoms (*B. jararaca* or *B. jararacussu* venom) were higher than IgG2a titers. IgE was not found in any of the four groups (data not shown).

**Figure 5:**
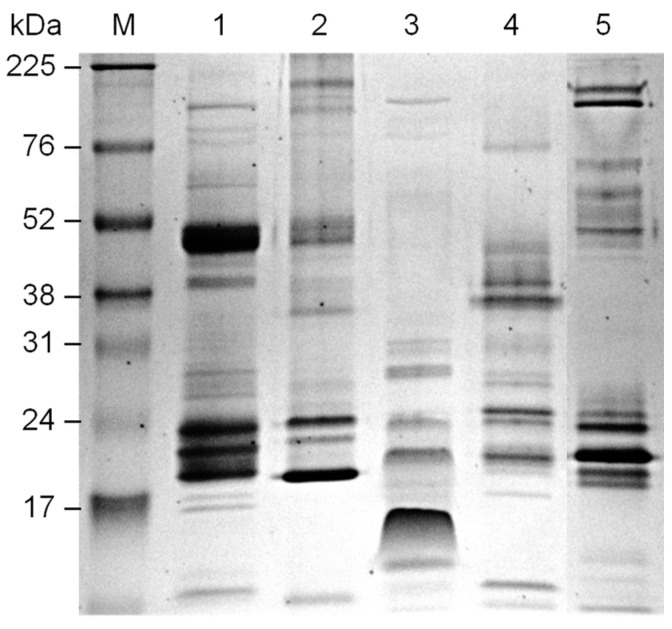
Electrophoretic profiles of snake venoms under non-reducing conditions. 12% SDS-PAGE of 5 µg of each snake venom. Coomassie blue staining. M: Rainbow marker high range (Cytiva); snake venom from 1: *B. jararaca*; 2: *B. alternatus*; 3: *B. jararacussu*; 4: *B. atrox amazonia*; 5: *Bitis anetans*.

Next, we evaluated the reactivity of antisera from mice subjected to oral tolerance with venoms of some *Bothrops* species and *Bitis anetans* by Western blotting. Protein profiles of the snake venoms under non-reducing conditions are shown in Figure 5; venoms differed in composition and band intensity.

**Figure 6:**
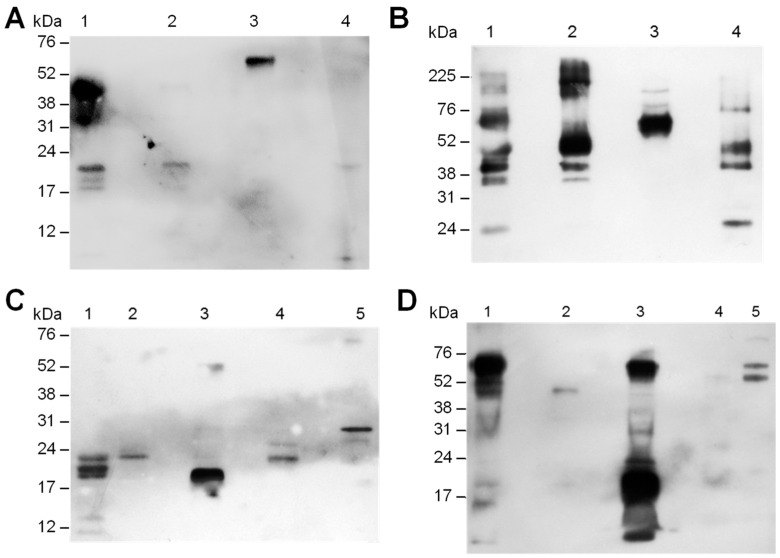
Analysis of the reactivity of the pool of antisera from animals subjected or not to oral tolerance induction with *B. jararaca* venom followed by immunization with snake venom. Snake venoms were subjected to electrophoresis and transferred to PVDF membrane according to Figure 5. The membrane was incubated with a pool of antisera corresponding to 45 days after immunization. (**A**): 1/400 dilution of Group I antisera; (**B**): 1/1000 dilution of Group II antisera; (**C**): 1/400 dilution of Group III antisera; (**D**) 1/1500 dilution of Group IV antisera. Peroxidase-conjugated anti-mouse IgG was then added, and the membrane was revealed with ECL reagent.

Snake venoms subjected to electrophoresis were transferred to PVDF membranes, the membrane was incubated with a pool of antisera corresponding to 45 days after the immunization of Groups I, II, III and IV and revealed by chemiluminescent reagent (Figure 6). Antisera from orally tolerized animals showed lower reactivity with snake venom proteins compared to antisera obtained from mice immunized with snake venom. Antisera of mice tolerized with *B. jararaca* venom and immunized with the same snake venom (Group I) recognized some proteins from the venom of *B. jararaca*, and a protein band from the venom of *B. alternatus*, *B. jararacussu* and *B. atrox amazonia* (Figure 6A). On the other hand, the antisera from mice immunized with the venom of *B. jararaca* recognized venom proteins of the *Bothrops* species analyzed (*Bothrops jararaca*, *B. alternatus*, *B. jararacussu* and *B. atrox amazonia*), and a wide range of reactivity was found for the venoms of *B. jararaca* and *B. alternatus* (Figure 6B). Interestingly, some protein bands recognized by both tolerized and non-tolerized mouse antisera (Groups I and II) were very similar (Figure 6A,B). When antisera from mice orally tolerized or not and immunized with *B. jararacussu* venom (Groups III and IV) were evaluated, at least one protein of the snake venom was recognized by antisera from Group III (Figure 6C), and antisera from immunized mice (Group IV) showed higher reactivity with *B. jararacussu* and *B. jararaca* venoms and low reactivity with other snake venoms (Figure 6D). The reactivity of Groups III and IV antisera was tested for *Bitis anetans* venom, and antisera from tolerized or not mice showed reactivity with this venom. Each antiserum recognized a distinct protein (Figure 6C,D). Our results showed that antisera from mice tolerized orally with *B. jararaca* venom and immunized with snake venoms had reactivity with venoms from other species at different levels. Interestingly, antisera from orally tolerized mice demonstrated cross-reactivity with different venom epitopes in comparison to the antisera of immunized animals.

## 3. Discussion

Oral tolerance is an immunological process in which the specific immune response is inhibited by prior oral administration of antigen. The induction of this process can be assessed after antigen immunization and measured by determination of antibody titer or by the decrease in allergic symptoms after allergen challenge [14].

The ability of the immune system to adapt to dietary antigens and commensal bacteria is important and prevents the development of food allergies, celiac disease, and autoimmune diseases [20]. Most studies related to oral tolerance have been carried out in animal models to establish the safe dose and duration of the process and to understand the immune cells and pathways involved, because of the risk of testing in humans [20]. These studies are of crucial importance for understanding the mechanisms involved and can promote strategies for the development of natural oral tolerance and prevent intoxication, allergies, and autoimmune diseases.

There is a report of daily oral administration for 14 days of *Crotalus durissus* terrificus snake venom (200 µg/kg) in male Swiss mice (17–21 g) that induced tolerance to the antinociceptive effect, and no antibody titers were measurable after prolonged treatment [21]. The expected effect was obtained by administering a dose of venom higher than that in our study, and mice were not immunized after receiving the oral dose. The cited study had a different purpose in relation to the present work and a different approach was taken.

Most incidences of snakebites in Brazil, considering all regions, are related to *Bothrops* species [22] and, therefore, constituted the objective of this study. They are also extensively investigated because they are responsible for more fatality cases in Central and South America than other groups of snakes [1]. So far, oral tolerance by administering *B. jararaca* venom in animal models has not been published and we have successfully established a protocol in BALB/c mice. The antibody titers were measured in the antisera after immunization with snake venoms to understand the mechanism involved.

We observed that mice receiving a single dose of 1.8 mg of *B. jararaca* venom orally did not develop tolerance and that the antibody titer was similar to that of the group of mice that was only immunized (Figure 1). These results are in agreement with those found in the literature for other antigens [11,16]. We have shown that repeated exposure to a dose of 1 µg of *B. jararaca* venom induced tolerance (Figure 1). We also found that tolerance was more effectively induced when animals received the same snake venom during oral administration and immunization (Figure 1, Figure 2 and Figure 3), showing that it is specific for one type of antigen, even when the antiserum showed cross-reactivity with venom from other snakes.

There was no reference concerning an immunization protocol with snake venom after oral administration, and we established that immunization would be performed about 7 days after the last dose. In the first protocol, mice were immunized 12 days after the last dose, while in the second protocol it was 8 days, and under both conditions, the oral tolerance induction was verified by low antibody titer. We think that the immunization protocol could be applied in this period of time to induce oral tolerance with *B. jararaca* venom.

In the first protocol of oral tolerance induction, mice received a low dose of *B. jararaca* venom and were immunized with *B. atrox* venom, and no tolerance induction was observed (Figure 1). On the basis of this result, we excluded this condition in the next protocol of oral tolerance induction and challenged the immunization with the venom of another *Bothrops* species (*B. jararacussu*). Partial oral tolerance induction was observed (Figure 2 and Figure 3) with *B. jararacussu* venom immunization, showing that this tolerance can be induced with another *Bothrops* venom. Variations in venom composition and biological activities of Brazilian snakes from *Bothrops* genus were observed [23] and these differences could be influenced on oral tolerance induction when immunization with heterologous venom was applied. Further studies involving different conditions for establishing oral tolerance induction with *B. jararaca* venom and other *Bothrops* species could elucidate these observations.

We also performed immunoblot analysis of the venom of different species of *Bothrops* incubating with antisera from animals tolerized with administration of *B. jararaca* venom or only immunized with snake venom. The epitopes recognized by each antiserum were clearly different (Figure 6), showing that the reactivity profile of antisera in relation to venom components changed according to the protocol used to induce tolerance. To see if proteins in snake venom from species other than *Bothrops* are recognized by antisera from mice orally tolerized or not, reactivity with *Bitis anetans* venom was evaluated. Antisera from mice partially tolerized (Group III) and from animals immunized with *B. jararacussu* venom (Group IV) were tested and each antiserum recognized different proteins in *Bitis anetans* venom, showing that reactivity with other snake venoms could be explored in future studies.

The application of oral tolerance has advantages because it is non invasive and uses a simple route. New knowledge and in-depth understanding of this process could contribute to the application of oral tolerance in prophylaxis and treatment of diseases [20].

Most of the studies related to oral tolerance induction involve the administration of one antigen [11,13,20]. The present work is an experimental study of oral tolerance induction developed with *B. jararaca* venom that is composed of a complex of proteins with biological activity. This process does not happen in the nature, and thus, the observation of oral tolerance induction represented a novel experimental approach. To demonstrate oral tolerance induction in our approach, the immune response was evaluated by the determination of the antibody titer, and then, the epitopes recognized by antiserum from orally tolerized or not tolerized mice were compared. Our results showed that it is possible to induce oral tolerance by administration of the complex mixture of the proteins, such as *B. jararaca* venom. Investigations related to the modulation of immune response and the mechanisms involved in this process were not part of the scope of the present work. The administration of a single high dose of *B. jararaca* venom was not lethal to the animals but did not induce oral tolerance. Further experimental studies should be conducted with other snake venoms to confirm this phenomenon as this approach would be used to develop vaccines by oral administration. The protocol established for oral tolerance induction should also be applied to other snake venoms in future studies together with the investigation of the mechanisms involved in this process. Therefore, the present work opened new perspectives to explore the process of oral tolerance induction.

## 4. Materials and Methods

### 4.1. Animals

BALB/c mice, female, aged 2–4 months, were used and maintained at the animal facilities of the Immunochemistry Laboratory, Butantan Institute, and they were caged and handled according to the International Animal Welfare Recommendations and in line with the guidelines for the use of animals in biomedical research [24]. Ethics Committee on Animal Use of the Butantan Institute approved the experiment protocol (Protocol IBU 454/08; 9 April 2008).

### 4.2. Snake Venoms

Lyophilized venoms (*Bothrops jararaca*, *B. alternatus*, *B. jararacussu*, *B. atrox amazonia* and *Bitis anetans*) were obtained from the Laboratory of Herpetology, Butantan Institute, São Paulo, Brazil, and stored at −20 °C. Venoms were resuspended in phosphate-buffered saline (PBS).

### 4.3. Protein Quantification

Protein concentration of the snake venom samples was determined by a microtiter-based Bradford Protein Assay (BioRad) in microplates using bovine serum albumin (BSA—Sigma–Aldrich, St. Louis, MO, USA) for the standard curve [25].

### 4.4. Induction of Oral Tolerance and Immunization

To establish the oral tolerance induction protocol, BALB/c mice were subdivided into 4 groups of animals (*n* = 3). Group I did not receive venom orally. Oral tolerance was induced by gavage administration of *B. jararaca* venom, and the mice received a single high dose of 1.8 mg (Group II) or 3 doses of 1 µg on the first, third and fifth day (Groups III and IV). At 12 days after the last low dose (Groups III and IV), all groups were intraperitoneally immunized with 1 µg of snake venom adsorbed to/encapsulated in nanostructured SBA-15 silica [26]. Groups I, II and III received *B. jararaca* venom, while Group IV was immunized with *B. atrox* venom. Antiserum corresponding to the 7th and 35th day after immunization was collected by bleeding from the retroorbital plexus and the antibody titer was determined by ELISA. Animals that did not undergo any procedure (*n* = 3) were considered the control group, and sera were added to the antibody titer assay.

Another oral tolerance induction protocol was then carried out. BALB/c mice were subdivided into 4 groups of animals (*n* = 5). Oral tolerance was induced by gavage administration of 1 µg of the *B. jararaca* venom on the first, third and fifth day (Groups I and III). Groups II and IV did not receive venom orally. Eight days after the last dose, mice were intraperitoneally immunized with 1 µg of snake venom adsorbed to/encapsulated in nanostructured SBA-15 silica. Groups I and II were immunized with *B. jararaca*, while Groups III and IV received *B. jararacussu* venom. Antiserum was collected by bleeding from the retroorbital plexus at 12, 25 and 45 days after immunization, and the antibody titers were determined by ELISA. Sera from mice of control group were also added. The cross-reactivity of serum after induction of oral tolerance with *B. jararaca* venom was analyzed by Western blotting.

### 4.5. ELISA Procedure

IgG titer of mouse sera was determined by ELISA. *B. jararaca* or *B. jararacussu* venom was diluted to 10 µg/mL in carbonate buffer (50 mM Na_2_CO_3_/NaHCO_3_, pH 9.6), and 100 µL/well were added to a 96-well EIA/RIA Clear Flat Bottom Polystyrene High Bind Microplate (3590, Corning, NY, USA). The plate was incubated at 4 °C overnight, washed 3 times with PBS/0.05% Tween and then incubated with the PBS/5% BSA blocking solution at 37 °C for 2 h. The blocking solution was replaced with a serial twofold dilution of antiserum in PBS/1% BSA, starting at an appropriate dilution for each one. Sera from animals that did not receive any venom was used as negative control, and samples were incubated at 37 °C for 1 h. Wells were washed 5 times with PBS/0.05% Tween to remove unbound antibodies. HRP-conjugated anti-mouse IgG (H + L) (Promega, Madison, WI, USA) was used as secondary antibody at 1/2500 dilution, and incubation was at 37 °C for 1 h. The plate was washed again, and the substrate used was OPD (o-phenylenediamine) (Sigma–Aldrich) in phosphate-citrate buffer with H_2_O_2_. After 5 min incubation, the reaction was stopped by adding 0.2 M citric acid. The absorbance at 450 nm was measured with a microplate reader. Antibody titers were expressed as log2 maximum serum dilution giving a positive reaction at which the absorbance was equal to two times the control value of the control serum.

Immunoglobulin isotypes, IgG1 and IgG2a, were evaluated in mouse antisera. For this purpose, HRP rat anti-mouse IgG1 (BD Biosciences, San Jose, CA, USA) at 1/400 dilution and HRP rat anti-mouse IgG2a (BD Biosciences) at 1/1000 dilution were used as secondary antibody. Antibody titer was determined as described above.

### 4.6. Electrophoresis and Western Blotting

Venom samples of 5 µg were prepared by adding non-reducing sample buffer and were separated by electrophoresis on 12% SDS-PAGE gel. Amersham ECL High-Range Rainbow Molecular Weight Markers (Cytiva, Marlborough, MA, USA) was also included. The gel was stained with Coomassie Blue or transferred to Amersham Hybond-P PVDF Membrane (Cytiva) for 1 h at 10 V using a BioRad Trans-blot SD semi-dry transfer cell. The membranes were stained with 0.5% Ponceau S, washed with ultrapure water and then blocked with PBS/5% BSA at 37 °C for 2 h. Next, the membranes were washed twice with PBS/0.1% Tween 20 for 20 s and were incubated overnight with an appropriate dilution of pooled antisera (Groups I, II, III and IV corresponding to 45 days after immunization) at 4 °C. The membranes were washed 4 times for 5 min and incubated with anti-mouse IgG (whole molecule)-HRP (Sigma) at 1/30,000 dilution in PBS/1% BSA for 1 h at room temperature. After washing, the blots were developed using Amersham ECL Prime Western Blotting Detection Reagent (Cytiva).

### 4.7. Statistical Analysis

Statistical analysis was performed by GraphPad Prism software. ANOVA (Tukey’s multiple comparison test) was used to compare average antibody titer obtained between antisera of animal groups, orally tolerized or not with *B. jararaca* venom. The sera obtained at different times after immunization were submitted to analyses. Two-way ANOVA with 95% confidence intervals was used to determine significant differences between IgG1 and IgG2a antibody titers for each mouse group.

## Data Availability

The data presented in this study are available on request from corresponding author.
